# Cell death signaling in human erythron: erythrocytes lose the complexity of cell death machinery upon maturation

**DOI:** 10.1007/s10495-025-02081-5

**Published:** 2025-02-09

**Authors:** Anton Tkachenko, Ondrej Havranek

**Affiliations:** 1 First Faculty of Medicine, BIOCEV, Charles University, Průmyslová 595, 25250 Vestec, Czech Republic; 2 First Department of Medicine – Hematology, General University Hospital and First Faculty of Medicine, Charles University, Prague, Czech Republic

**Keywords:** Apoptosis, Cell death, Ferroptosis, Necroptosis, Red blood cell

## Abstract

Over the recent years, our understanding of the cell death machinery of mature erythrocytes has been greatly expanded. It resulted in the discovery of several regulated cell death (RCD) pathways in red blood cells. Apoptosis (eryptosis) and necroptosis of erythrocytes share certain features with their counterparts in nucleated cells, but they are also critically different in particular details. In this review article, we summarize the cell death subroutines in the erythroid precursors (apoptosis, necroptosis, and ferroptosis) in comparison to mature erythrocytes (eryptosis and erythronecroptosis) to highlight the consequences of organelle clearance and associated loss of multiple components of the cell death machinery upon erythrocyte maturation. Recent advances in understanding the role of erythrocyte RCDs in health and disease have expanded potential clinical applications of these lethal subroutines, emphasizing their contribution to the development of anemia, microthrombosis, and endothelial dysfunction, as well as their role as diagnostic biomarkers and markers of erythrocyte storage-induced lesions. Fas signaling and the functional caspase-8/caspase-3 system are not indispensable for eryptosis, but might be retained in mature erythrocytes to mediate the crosstalk between both erythrocyte-associated RCDs. The ability of erythrocytes to switch between eryptosis and necroptosis suggests that their cell death is not a simple unregulated mechanical disintegration, but a tightly controlled process. This allows investigation of eventual pharmacological interventions aimed at individual cell death subroutines of erythrocytes.

## Introduction

Cell death is one of the most fundamental properties of all living organisms, playing an indispensable role in many biological processes including evolution of life. Mounting evidence suggests that regulated cell death (RCD), which is defined as a controlled and tightly regulated self-destruction of cells, dates back to the earliest prokaryotes [1]. This is in contrast with the widely recognized hypothesis that cell death evolved as one of the cooperation mechanisms between cells in multicellular organisms (the multicellularity-cooperation hypothesis) [2]. Another hypothesis explaining the origin of RCDs links RCD development to the adaptation to autonomous DNA molecules like plasmids (the addiction hypothesis) [3]. Finally, the pleiotropy-original sin hypothesis postulates that cell death is an immanent and inevitable process associated with the formation of metabolic by-products in cells like reactive oxygen species (ROS) and cell death machinery components-encoding genes were not initially related to cell death processes and performed pro-survival functions. However, they promoted cell death under some unfavorable conditions [2, 4]. Alternatively, RCD might have originated as an anti-viral mechanism (the viral defense-immunity hypothesis) [5]. Despite the lack of the consensus concerning the exact origin of RCD in the research community, recent advances in our understanding of cell death evolution indicate that multiple forms of RCDs (summarized and defined by the Nomenclature Committee on Cell Death, NCCD) [6] co-evolved. This co-evolution of multiple forms of RCD explains the multilevel and wide crosstalk between them and facilitates a change in our view of individual and separate RCD modalities (like apoptosis, autophagy-related cell death, necroptosis, pyroptosis, ferroptosis and others) towards the concept of a tightly interconnected and complex RCD network [7, 8]. Multiple pathways are shared by different RCDs and certain signaling components act as molecular switches or represent cell fate decision points with multiple backup functions within the network [9]. Intriguingly, accumulating evidence indicates that cell death signaling can be cell type-specific. For instance, entosis occurs in epithelial cells [10], pyroptosis is typical for macrophages [11], and neutrophils frequently undergo NETotic cell death, generating neutrophil extracellular traps (NETs) to immobilize pathogens [12]. On the other hand, the key factors that determine cell fate decisions in particular cell types are not fully understood. Identification of such cell type-specific cell death pathways that determine a particular cell death mode might lead to establishment of cell type-selective therapeutic targets.

More and more studies report that mammalian erythrocytes can experience senescence, apoptosis, and necroptosis [13–18]. Despite a general resemblance to their counterparts in nucleated cells, the apoptotic and necroptotic signaling pathways in erythrocytes have certain critical differences. To reflect that, specific terms were introduced to describe apoptosis- and necroptosis-like cell deaths in enucleated cells: *eryptosis* and *erythronecroptosis*, respectively. The major differences between nucleated and enucleated cells regarding these two RCD modes are summarized in two recently published articles [18, 19]. Briefly, eryptosis does not rely on caspase-3 as an indispensable executioner, but primarily requires elevation of intracellular Ca^2+^ levels. Despite the importance of reactive oxygen species (ROS) signaling in both eryptosis and apoptosis, apoptosis is associated with a broader network of ROS signaling [17]. Paradoxically, evidence shows that casein kinase 1α (CK1α), protein kinase C (PKC), and Janus kinase 3 (Jak3) activate eryptosis, but mostly inhibit apoptosis [19]. Necroptosis of erythrocytes relies on the RIPK1 (receptor-interacting serine/threonine-protein kinase 1)/RIPK3 (receptor-interacting serine/threonine-protein kinase 3)/MLKL (mixed lineage kinase domain-like pseudokinase), similarly to nucleated cells. Unexpectedly, erythronecroptosis is not triggered by tumor necrosis factor-α (TNF-α) or TNF-related apoptosis-inducing ligand (TRAIL) known as strong inducers of necroptosis in nucleated cells [18]. However, it is not known what the cause is and what are the underlying factors responsible for these observed discrepancies between mature erythrocytes and nucleated cells. As outlined above, the intracellular cell death apparatus monitors pro-survival and cell death-promoting inputs to provide an informed decision regarding the cellular fate. At the level of subcellular compartments, such as the nucleus, mitochondria, lysosomes, endoplasmic reticulum (ER), Golgi apparatus, etc., these perturbations are sensed and activate the corresponding signal transduction pathways to generate the appropriate biological response. Thus, nuclear, mitochondrial, lysosomal, reticular, and other organelle-associated signaling and executive pathways are critical for RCD initiation and execution [20]. Maturation of erythrocytes during erythropoiesis results in expulsion of the majority of organelles resulting in the formation of mature erythrocytes devoid of organelles [21] critically involved in cell death signaling. What happens to the cell death machinery of erythrocytes upon maturation? In this review, we summarize the currently available data regarding RCD machinery of erythroid precursors and mature erythrocytes to provide evidence that the limited set of RCDs in mature erythrocytes is mainly associated with the organelle clearance from erythrocyte precursors.

## Erythropoiesis: a brief overview of its steps and regulation

Erythron comprising circulating erythrocytes and their precursor cells in the bone marrow provides a critical function of oxygen transport [22]. On top of that, multiple studies have shown that erythron is involved in additional functions. These include regulation of the systemic metabolic control [23], fine-tuning of the endothelial function via nitric oxide (NO) scavenging and NO bioavailability adjustment [24, 25], modulation of innate immunity (acting as immune sentinels) [26], regulation of cytokine and chemokine levels in circulation [27], and production of ROS via hemoglobin- or heme-mediated pathways (as a part of the immune response to pathogens) [28].

Traditionally, erythropoiesis is defined as a gradual, stepwise, hierarchical and unidirectional process that starts from hematopoietic stem cells (HSCs) residing in the bone marrow (in adults) and extramedullary sites, which are precursors for all types of blood cells [29]. Extramedullary erythropoiesis occurs primarily in spleen and liver and contributes to RBC production in stress conditions or disease [30]. However, recent advances in our understanding of hematopoiesis in general suggest that this process might be of a more complex nature with different alternative routes and bypasses [31, 32]. HSCs are differentiated into common myeloid progenitors (CMPs), which give rise to the entire myeloid lineage [33]. Further differentiation of CMPs results in formation of megakaryocytic-erythroid progenitors (MEPs), acting as precursors for both erythrocytes and platelets [32]. Notably, MEP downstream bifurcation into megakaryocytic or erythroid lineage is at least partly driven by the cell cycle regulation [34]. Additionally, MEP fate decision leading to erythroid lineage differentiation requires MYB and extracellular signal-regulated kinase (ERK) activation [35]. MEPs consecutively differentiate into erythroid burst-forming units (BFU-e) and erythroid colony-forming units (CFU-e), the first exclusively erythroid progenitors [31]. In contrast to CFU-e, BFU-e progenitors proliferate slowly [36] and respond to stem cell factor (SCF), interleukin-3 (IL-3), IL-6, and insulin-like growth factor 1 (IGF-1) [36–38]. It is important to mention that expansion of the erythroid progenitors occurs primarily at the level of rapidly dividing CFU-e in erythropoietin (EPO)-dependent manner [39]. EPO acts as a master regulator of erythropoiesis. It is produced by EPO-synthesizing renal tubular interstitial cells in response to hypoxia. Additionally, up to 10–15% of EPO is produced in liver, adjusting the proportion based on requirements [40]. Upon binding to the erythropoietin receptor (EpoR, located on the surface of erythroid progenitor cells), EPO activates Jak2/STAT5, Ras/Raf/MEK/ERK and PI3K/Akt signaling pathways [41, 42] with consequent downstream recruitment of GATA-1 transcription factor [43], a master regulator of erythropoiesis critically affecting all aspects of cell survival and proliferation. Most importantly, GATA-1 upregulates autophagy-related and cell cycle-regulating genes, pro-survival anti-apoptotic Bcl-2, and downregulates myeloid genes to ensure the maintenance of erythroid specification [44].

The next stage of erythropoiesis includes differentiation of BFU-e and CFU-e progenitors into proerythroblasts [45] with their further consecutive conversion into basophilic, polychromatic and orthochromatic erythroblasts [45]. At this stage, erythropoiesis occurs within a specialized microenvironment, the so-called erythroblastic islands comprising of a centrally located macrophages which are surrounded by erythroblasts [46, 47]. Erythroblastic islands-associated tissue-resident ferritin-enriched macrophages perform a function of erythroblast nurse cells, supporting their survival and maturation, providing iron supply for hemoglobin synthesis, promoting proliferation via secreting erythropoiesis-enhancing factors such as IGF-1 and IL-18, and ensuring elimination of phosphatidylserine-exposing pyrenocytes, which represent the extruded nuclei formed within the enucleation process [47–49]. Notably, clearance of organelles occurs at the erythroblastic and reticulocytic stages of erythropoiesis. After nucleus expulsion, nucleus-free reticulocytes are released into the bloodstream and pyrenocytes are engulfed by central macrophages of the erythroblastic islands via phosphatidylserine-mediated “eat-me” signals [50]. This process relies on KLF1 (Krueppel-like factor 1), FOXO3 (forkhead box O3), and E2F2 transcription factors, chromatin condensation-regulating caspase-3, DNA methylation-regulating Tet family proteins, Asxl1, cytoskeleton-regulating Rho GTPases, and p38 MAPK signaling [51, 52].

Enucleated reticulocytes lose their organelles to become mature erythrocytes, superspecialized cells deprived of organelles to accommodate more oxygen-transporting hemoglobin. Given the importance of mitochondria as a central organelle involved in the control of RCDs [53, 54], it is important to highlight how clearance of mitochondria occurs in the erythroid lineage. Mitochondria are eliminated at the stage of erythroblasts and reticulocytes via either macroautophagy (together with other organelles) or mitophagy, which is a more selective process [55]. Of note, Atg5/Atg7-dependent macroautophagy occurs predominantly at the level of early erythroblasts with mitophagy taking place during further erythroblast differentiation [56]. On the other hand, ATG4a-dependent macroautophagy seems to contribute to mitochondrial clearance in late erythroblasts as well as in reticulocytes [57]. A growing body of evidence indicates that the PINK1/Parkin pathway [56] and BNIP3L/NIX signaling [58, 59] are involved in mitochondrial clearance in the erythroid lineage as well. Moreover, voltage-dependent anion-selective channel protein 1 (VDAC1)-mediated mitophagy has been also reported [55]. Importantly, inefficient elimination of mitochondria in red blood cell (RBC) precursors of sickle-cell disease patients results in mitochondrial retention in mature erythrocytes with consequent increased ROS generation and accelerated hemolysis [60, 61].

Like mitochondria, lysosomes are important regulators of cell death pathways. They can act as a quality control, triggering or amplifying signals for diverse RCDs [62–64]. The process of lysosomes elimination during erythroblasts differentiation is much less studied in comparison to mitochondrial clearance. In human erythropoiesis, evidence suggests that ATG4b-dependent autophagy is required for lysosome elimination [65]. Additionally, the mDia2/Chmp5 pathway likely contributes to the autophagosome-lysosome fusion and lysosomal clearance in the erythrocyte precursor cells [66]. At the same time, it has been hypothesized that autophagy pathways involved in lysophagy in non-erythroid cells, e.g., the p62-mediated pathway [67], might be responsible for the clearance of lysosomes in the erythroid lineage as well [51]. We believe that further studies are needed to fully understand mechanisms of lysosome elimination in the erythroid-committed precursors.

Following enucleation, the newly formed reticulocytes known as R1 reticulocytes are larger in comparison to mature erythrocytes and still contain residual organelles. During their conversion to R2 reticulocytes, which are consequently released into the bloodstream, R1 reticulocytes lose excess plasma membrane. Additional loss of plasma membrane results in the formation of R3 reticulocytes [68]. During their maturation, reticulocytes continuously lose mitochondria with gradual reduction of their mitochondrial content. It has been even proposed that mitochondrial content could serve as a method to distinguish different stages of reticulocytes maturation [69]. Further membrane and proteome remodeling, as well as organelle clearance, occurs directly in the bloodstream and results in the formation of mature erythrocytes [70].

In this review, we focus on features of RCDs during erythropoiesis. Specifically, we describe the differences between erythrocyte precursors and mature erythrocytes and how erythrocyte maturation results in retaining the less branched cell death signaling network due to the loss of cellular structural complexity.

## The cellular complexity-related features of apoptosis: involvement of organelles

Apoptosis is a caspase-dependent RCD which was first described in 1972 based on a characteristic cellular morphology such as cell shrinkage, nuclear condensation, formation of apoptotic bodies, etc [71, 72]. According to the NCCD, the key determinants that define apoptosis as an RCD are its initiation by caspase-8 and caspase-9 and cell death execution by caspase-3, caspase-6, and caspase-7, which occur at the terminal stage when the cells are already committed to die [6, 73]. Apoptotic pathway is the most studied type of RCD. Its contribution to embryonic development, maintenance of homeostasis, as well as involvement in other basic physiological processes and pathological situations is well documented [73]. Apoptosis is also successfully targeted pharmacologically. For example, a selective pro-apoptotic Bcl-2 inhibitor Venetoclax has been approved for the treatment of chronic lymphocytic and acute myeloid leukemia [74].

Generally, apoptosis can be executed via two core pathways: extrinsic and intrinsic [75, 76]. The extrinsic apoptosis pathway is triggered by death receptor-death ligand interactions, including FasL/FasR, TRAIL/TRAIL-R1 or TRAIL-R2, and TNF/TNF-R1 [77–79]. Upon binding of a ligand to a death receptor, Fas-associated death domain protein (FADD) is recruited and the death-inducing signaling complex (DISC) (for FasL/FasR, TRAIL/TRAIL-R1 or TRAIL-R2) or complex II (in the case of TNF/TNF-R1) is formed. DISC consists of FADD, pro-caspase-8 and the cellular FLICE inhibitory proteins (c-FLIPs). Its formation results in activation of initiator caspase-8 with further downstream recruitment of executioner caspase-3 by limited proteolysis [73, 75]. In contrast to the extrinsic pathway, the intrinsic pathway is driven mainly by intracellular stimuli and mediated primarily by the mitochondrial outer membrane permeabilization (MOMP) triggered by pro-apoptotic Bax and Bak proteins [80]. MOMP results in the release of proteins residing in the mitochondrial intermembrane space (e.g., cytochrome c and Smac) [81]. Cytochrome c binds to the apoptotic peptidase activating factor 1 (Apaf-1), forming an apoptosome with consequent downstream activation of caspase-9 and caspase-3. At the same time, Smac (second mitochondria-derived activator of caspase) inactivates caspase-inhibiting X-linked inhibitor of apoptosis protein (XIAP), which functionally contributes to the recruitment of caspase-3 and caspase-7 [82]. Of note, the role of mitochondria in apoptosis is not limited to the intrinsic pathway. Compelling evidence suggests that mitochondria are downstream effectors of the death receptors-mediated extrinsic apoptotic pathway via t-Bid-mediated MOMP [83]. This clearly suggests that MOMP is critical for survival/apoptosis balance regulation in eukaryotic cells.

Ample evidence suggests that lysosomes have been implicated in multiple RCD subroutines primarily through lysosomal membrane permeabilization (LMP) and subsequent release of cathepsins [84]. It has been shown that multiple apoptosis-regulating proteins are substrates for lysosome-derived cathepsins, including Bid, Bcl-2, Bcl-XL, and XIAP [62]. Conversely, mitochondria-derived ROS and pro-apoptotic proteins like Bax, Bid or Bim can trigger LMP, while caspase-8 regulates release of cathepsins [85]. This suggests an extensive crosstalk between mitochondria and lysosomes in apoptosis [86].

ER stress caused by accumulation of unfolded or misfolded proteins with consequent induction of compensatory unfolded protein response is another important trigger of apoptosis [87, 88]. Individual components of the apoptotic machinery involved in regulation of ER stress-mediated cell death include Bax, Bak, Bim, or PUMA (p53 upregulated modulator of apoptosis), as well as the transcription factor CHOP (C/EBP homologous protein)-dependent pathway [89]. Similarly to lysosomes, the interplay between ER and mitochondria is very important for apoptosis regulation. The ER/mitochondrial crosstalk is provided by involvement of Bax, Bak, Bim, or PUMA, as well as MOMP-regulating Ca^2+^ signaling and communication through mitochondria-associated ER membranes (MAMs) [90, 91].

The Golgi apparatus hosts various components of the pro- and anti-apoptotic machinery, which indicates that it might be capable of sensing stress signals and thus contribute to the change in the cell fate direction [92]. Indeed, an increasing body of evidence demonstrates that the Golgi apparatus regulates apoptosis signaling via the Golgi apparatus-associated proteins like P115, GRASP65, Bruce, and Golgi anti-apoptotic protein (GAAP). For instance, P115 and GRASP65 trigger apoptosis via the ERK/p53/PUMA pathway or Fas-mediated signaling, respectively, whereas Bruce and GAAP decrease apoptosis via caspase-9, Smac and p53 inhibition, and Ca^2+^ signaling downregulation [93].

Morphological changes in the nucleus associated with apoptosis such as karyopyknosis and karyorrhexis have been described in early studies. However, accumulating data indicate that the nucleus is also involved in regulation of apoptosis [94, 95]. Notably, apoptosis is triggered in response to DNA damage resulting in nuclear p53 activation and consequent upregulation of pro-apoptotic proteins and their translocation to mitochondria to trigger the MOMP-mediated pathway. Notably, pleiotropic p53 affects the extrinsic as well as intrinsic apoptotic pathway [94]. Furthermore, the nuclear envelope also affects apoptosis. It separates the nucleus from the cytosol and changes in its selective permeability mediate leakage of caspases and other pro-apoptotic factors into the nucleus. On the other hand, the nuclear envelope is not just a passive target for caspases that cleave its structural proteins to increase nuclear permeability. In particular, the active participation of the nuclear envelope in apoptosis includes generation of nuclear protein-containing nuclear bubbles (GRUNB) in a Bax-dependent way and their rupture may amplify apoptotic signaling. Furthermore, the nuclear envelope acts as a platform where apoptotic complexes might be assembled [95].

Taken together, the wide complexity of apoptotic network, including involvement of cellular organelles, is well-established. Overall, it ensures adequate cell fate decision-making in response to multiple frequently multidirectional external and internal signals.

## Apoptosis in erythrocyte precursors

A capability to execute intrinsic and extrinsic apoptosis is widely documented in the erythroid lineage at different stages of its development and differentiation [96–98]. Moreover, apoptosis seems to play an indispensable role in erythropoiesis regulation [99]. This role is mediated by multiple cytokines, growth factors and death receptor-ligand systems that execute their effect on erythropoiesis partially through apoptosis modulation. These include EPO, TNF-α, IFN-γ (interferon-γ), IL-3, SCF, Fas/FasL system, or TRAIL signaling [100–103].

**Fig. 1:**
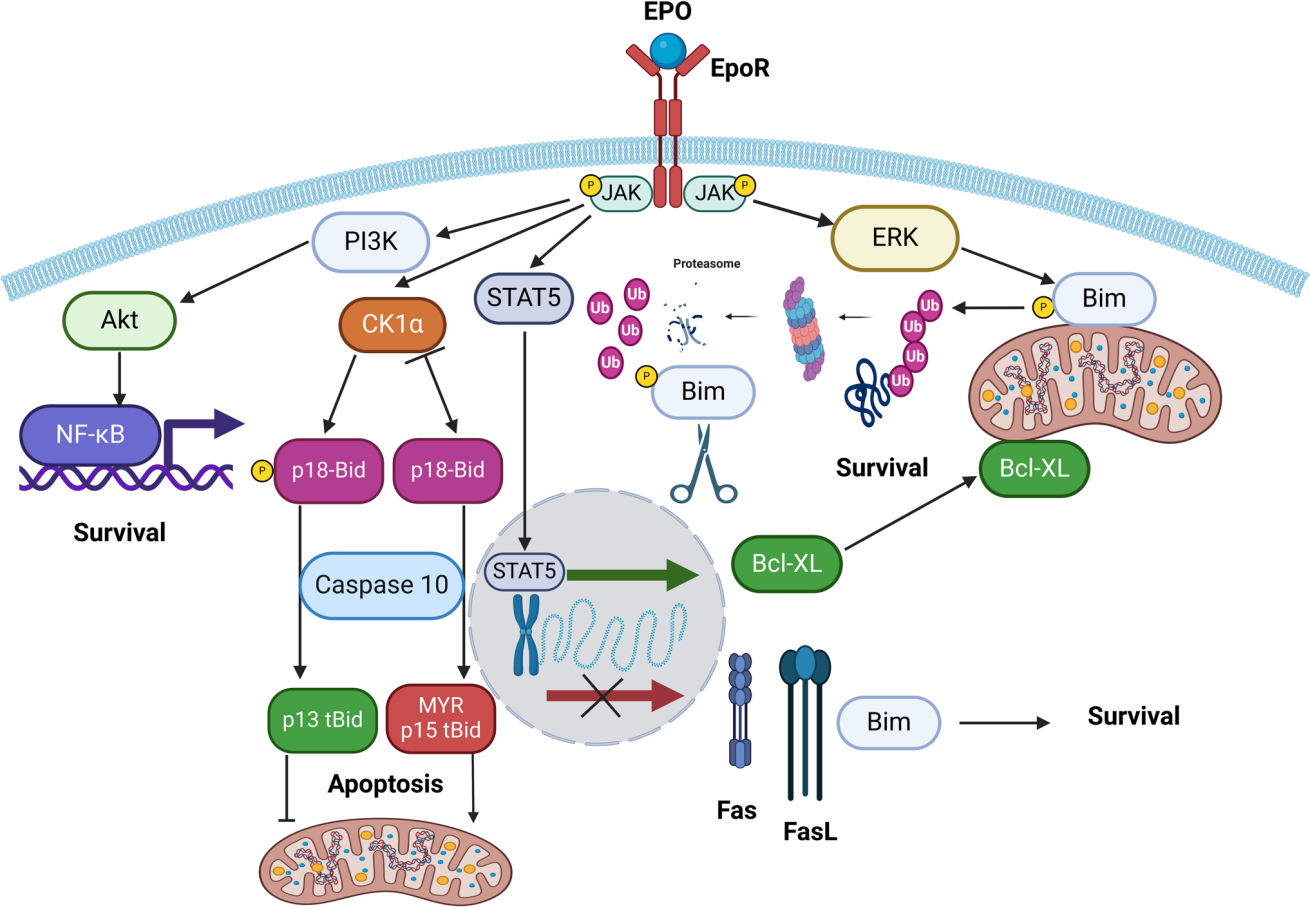
Anti-apoptotic EPO signaling in the erythroid lineage. EPO/EpoR signaling in erythroid precursors promotes survival and inhibits apoptosis through PI3K/Akt signaling, JAK/STAT5-mediated upregulation of anti-apoptotic Bcl-XL and downregulation of pro-apoptotic Fas, FasL, or Bim, CK1α-dependent p18-Bid phosphorylation, or ERK-dependent Bim phosphorylation with its further proteasome-dependent degradation. *Akt* – protein kinase B; *Bid* – BH3-interacting domain death agonist; *Bcl-XL* – B-cell lymphoma-extra large; *Bim* – Bcl-2 interacting mediator of cell death; *CK1α* – casein kinase 1 α; *EPO* – erythropoietin; *EpoR* – erythropoietin receptor; *ERK* – extracellular signal-regulated kinase; *JAK* – Janus kinase; *NF-κB* – nuclear factor kappa-light-chain-enhancer of activated B-cells; *P* – phosphate group; *PI3K* – phosphoinositide 3-kinase; *STAT* – signal transducers and activators of transcription; *Ub* – ubiquitin

Anti-apoptotic effects of EPO (targeting primarily CFU-e and proerythroblasts) are mediated via pro-survival PI3K/Akt signaling activation [104], JAK2/STAT5-dependent upregulation of anti-apoptotic Bcl-XL [105, 106], JAK2/STAT5-dependent downregulation of pro-apoptotic Fas, FasL and BH3-only protein Bim [103], activation of the CK1α-dependent caspase-10-P13tBid axis [107], and ERK-dependent degradation of Bim [108], summarized in Fig. 1. Importantly, EPO prevents Fas/FasL-mediated apoptosis in erythroblasts [109] and CFU-e cells [31]. Similarly, the anti-apoptotic effect of SCF and IL-3 on erythroid precursors is also mediated by PI3K/Akt and JAK/STAT signaling pathways [102, 110]. Expression of Fas and FasL depends on the stage of erythropoiesis, which allows regulating expansion of cells via inducing apoptosis of Fas-expressing immature erythroblasts by FasL-expressing mature erythroblasts [98]. Thus, immature erythroblasts are vulnerable to Fas/FasL-induced caspase-8-mediated extrinsic apoptosis, which is a physiological mechanism that regulates erythropoiesis [21, 107]. Additionally, Fas/FasL signaling is involved in TNF-α- and IFN-γ-mediated apoptosis in the erythroid lineage [101]. SCF was shown to inhibit Fas-mediated apoptosis in CFU-e [111]. Similar to the Fas/FasL system, TRAIL expressed in mature erythroblasts can promote apoptosis of TRAIL-R1- and TRAIL-R2-expressing immature erythroid cells [98]. TRAIL-R2 was demonstrated to be more involved in erythropoiesis [112].

Apoptosis is also active in reticulocytes. It has been shown that pro-survival MOMP-inhibiting Bcl-XL is essential to protect reticulocytes from apoptosis and that its loss results in severe impairment of erythropoiesis and anemia development [113]. Bcl-XL accumulation occurs specifically at the erythroblastic stage of differentiation before enucleation [114]. The levels of Bcl-XL decrease with mitochondrial clearance during reticulocyte maturation together with its deamidation [115]. Of note, Bcl-XL deficiency-associated reticulocyte apoptosis does not rely on pro-apoptotic Bim and PUMA [113]. At the same time, bone marrow-derived nascent reticulocytes are less susceptible to the cytochrome c-mediated intrinsic apoptosis due to the lack of caspase-9 and low levels of Bax, Apaf-1, and pro-caspase-3 [115]. Moreover, the apoptotic machinery itself regulates mitochondrial clearance during terminal erythropoiesis, e.g., Nix protein mediates autophagy-dependent mitochondrial elimination in reticulocytes [116]. Erythroid terminal differentiation seems to be promoted also by alternative EPO function. A recently published study showed that EPO activated CK1α, which prevented MYR-P15-tBid-associated apoptosis and generated pro-survival P13-tBid in a caspase-10-mediated fashion [107]. Furthermore, this study supplements an earlier hypothesis suggesting that Bid cleavage is an important switch between apoptosis and further erythroid lineage differentiation [98, 117]. Accumulating evidence suggests that caspases play a multilevel role in erythropoiesis - balancing cell death, differentiation, and clearance of organelles [98, 118]. Notably, in erythroid cells, caspase-3 activation does not guarantee apoptosis execution, since the transcriptional factor GATA-1 essential for erythropoiesis is protected from caspase-3 by HSP70 (heat shock protein 70). Thus, caspase-3 performs non-apoptotic functions [119]. Caspase-3 is known to promote differentiation and expansion of erythroblasts [120].

Importantly, apoptosis of erythroid cells can be fine-tuned in stress conditions to ensure adequate erythropoiesis. In particular, pro-apoptotic Noxa is dispensable for normal erythropoiesis. However, it triggers apoptosis in erythroid precursors in the case of cytokine deprivation to prevent expansion of erythroblasts and to control the number of erythrocytes under stress conditions [99].

It is unfortunate that apoptotic involvement of lysosomes, ER, and the cell nucleus in differentiation of erythroid cells is mostly beyond the focus of most studies. Nevertheless, lysosomes seem to contribute to the anti-apoptotic effect of EPO. For instance, survival of maturing erythroblasts requires *Serpina3g*/Spi2A-dependent inhibition of lysosomal cathepsins B/L [121]. Notably, following internalization, EPO/EpoR complexes are degraded in lysosomes [122]. ER involvement in the regulation of apoptosis is executed by levels of Ca^2+^ leakage from ER, which is regulated by Bcl-2 [123]. Furthermore, DNA methyltransferase 1 (DNMT1) protects erythroblasts from p53/caspase-3 pathway-dependent apoptosis through reducing ER stress [124]. Additionally, Fas-mediated apoptosis of erythroid precursors in low grade myelodysplastic syndromes is linked to ER via caspase-8-mediated cleavage of B-cell receptor-associated protein 31 (BAP31), which is an ER-associated chaperone and quality control protein. Under these circumstances, BAP31 is cleaved with consequent formation of the p20 fragment, which activates the mitochondrial apoptotic events such as cytochrome c release, resulting in activation of the intrinsic apoptotic pathway [123, 125]. There are still a lot of unanswered questions concerning the crosstalk between organelles in apoptosis during erythropoiesis.

**Fig. 2:**
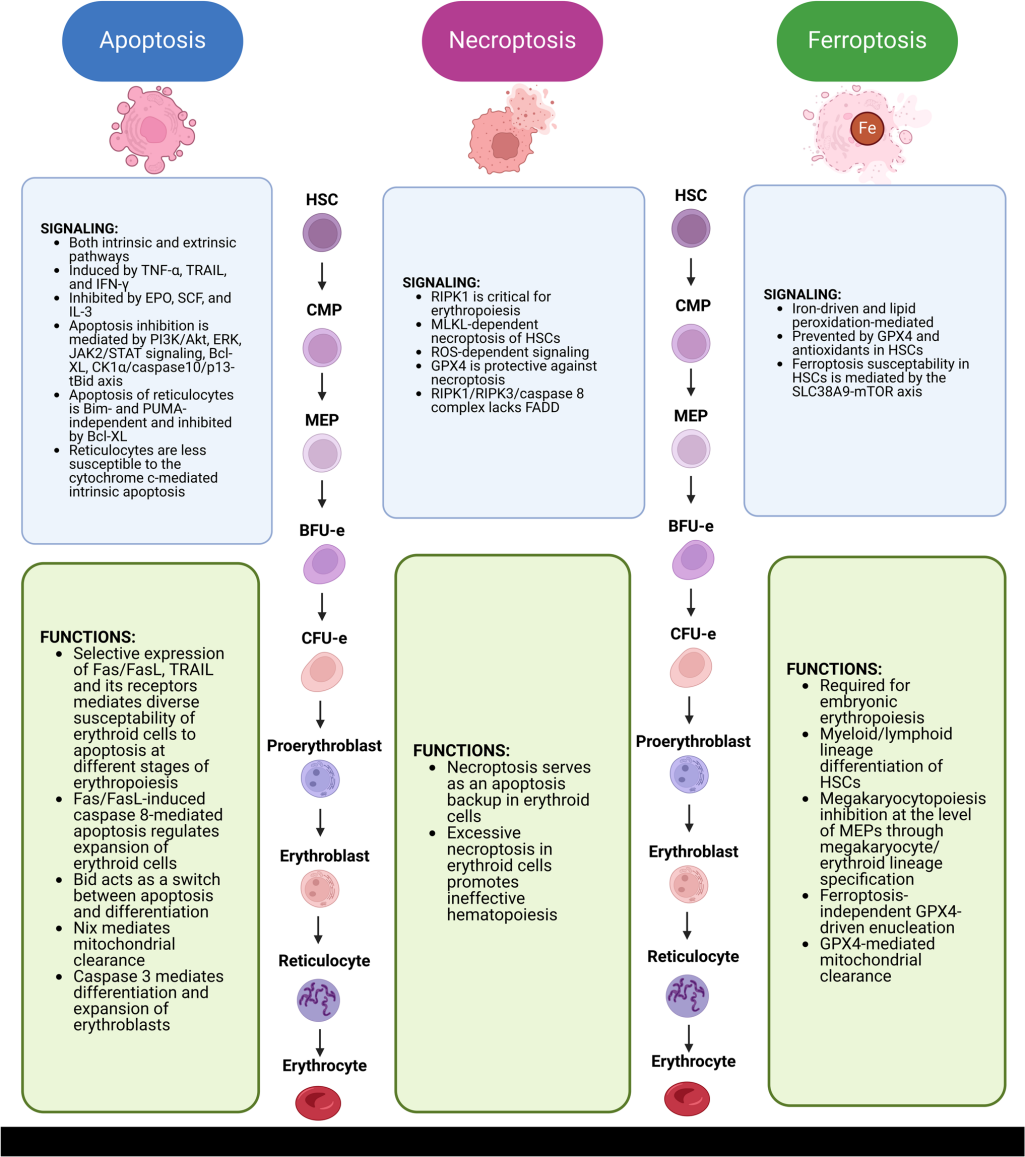
An overview of cell death pathways and their individual components in the erythroid lineage: apoptosis, necroptosis, and ferroptosis with an emphasis on the role in erythropoiesis. Erythroid cells can undergo apoptosis, necroptosis, and ferroptosis, which are involved in regulation of erythropoiesis under physiological and pathological circumstances. *Akt* – protein kinase B; *BFU-e* – burst-forming unit-erythroid; *Bid* – BH3-interacting domain death agonist; *Bcl-XL* – B-cell lymphoma-extra large; *Bim* – Bcl-2 interacting mediator of cell death; *CFU-e* – colony-forming unit-erythroid; *CMP* – common myeloid progenitor; *CK1α* – casein kinase 1 α; *FADD* – Fas-associated protein with death domain; *EPO* – erythropoietin; *ERK* – extracellular signal-regulated kinase; *GPX4* – glutathione peroxidase 4; *HSC* – hematopoietic stem cells; *IFN-γ* – interferon γ; *IL-3* – interleukin 3; *JAK* – Janus kinase; *MEP* – megakaryocytic-erythroid progenitor; *mTOR* – mammalian target of rapamycin; *MLKL* – mixed lineage kinase domain like pseudokinase; *PI3K* – phosphoinositide 3-kinase; *PUMA* – p53 upregulated modulator of apoptosis; *RIPK1* – receptor-interacting serine/threonine-protein kinase 1; *RIPK3* – receptor-interacting serine/threonine-protein kinase 3; *ROS* – reactive oxygen species; *SCF* – stem cell factor; *SLC38A9* – solute carrier family 38 member 9; *STAT* – signal transducers and activators of transcription; *TNF-α* – tumor necrosis factor α; *TRAIL *– tumor necrosis factor (TNF)-related apoptosis-inducing ligand

To sum up, apoptosis of erythroid cells plays an important role in erythropoiesis and its regulation (Fig. 2). Erythropoiesis depends on the balance between anti-apoptotic (EPO, IL-3, and SCF) and pro-apoptotic (Fas/FasL, TRAIL, and TNF-α) signaling, primarily the EPO/Fas-FasL ratio. Furthermore, expression of pro-apoptotic and anti-apoptotic proteins is variable during individual stages of erythropoiesis, which determines apoptosis susceptibility at each stage of differentiation. In particular, the pro-apoptotic phenotype is switched to the anti-apoptotic one at the level of proerythroblasts [126]. Bid cleavage and involvement of caspases might not only promote apoptosis, but, on the contrary, mediate survival and differentiation of erythroid cells. Taken together, multiple components of the apoptotic machinery participate in the differentiation of erythroid cells.

## The cellular complexity-related features of necroptosis: contribution of organelles

Necroptosis (a regulated necrosis) is a distinct lytic type of RCD controlled by the RIPK1/RIPK3/MLKL axis. It is triggered primarily by Fas, TNF-α, and TRAIL death receptors signaling [127]. Necroptosis culminates in activation of MLKL with its consequent oligomerization and formation of cell membrane pores. This determines the strongly pro-inflammatory characteristics of this RCD with poorly controlled inflammatory response [128]. Extensive crosstalk between apoptosis and necroptosis is well-documented and is mediated primarily by the caspase-8 status (pro-apoptotic, anti-necroptotic), post-translational modifications of RIPK1, RIPK3 and MLKL (phosphorylation and ubiquitination), features of ROS signaling, etc [129, 130]. Converging lines of evidence indicate that pro-inflammatory necroptosis is an important pathogenetic factor in multiple diseases, which is summarized in several recent review articles [131–133].

Apart from the above-mentioned canonical necroptosis activation, there is abundant evidence that necroptosis is tightly associated with mitochondrial dysfunction [134]. Some studies and experimental data support the existence of mitochondria-independent necroptotic pathways and the ability of cells to undergo necroptosis following mitophagy-mediated depletion of mitochondria [135, 136]. However, a growing body of evidence suggests that mitochondria are critically involved in necroptosis initiation and execution, including mitROS-mediated RIPK1 and RIPK3 recruitment, feedback RIPK1/RIPK3-mediated mitROS generation, RIPK3-dependent pyruvate dehydrogenase complex-mediated ROS production, or phosphoglycerate mutase family member 5 (PGAM5)-dependent dynamin-related protein 1 (Drp1)-mediated necroptosis (PGAM5 is activated downstream of the RIPK1/RIPK3/MLKL complex) [137–140].

Importantly, Ca^2+^- and ROS-dependent LMP has been reported to contribute to necroptosis via release of cathepsins, cathepsins-mediated mitochondrial dysfunction, and lysosome-dependent regulation of RIPK1 and RIPK3 post-translational modifications [141]. Moreover, LMP can occur downstream of MLKL recruitment, suggesting that lysosomes participate in execution of necroptosis [142].

Accumulating evidence supports the role of ER in regulation of necroptosis. For instance, ER stress has been shown to trigger RIPK1/RIPK3/MLKL-dependent necroptosis [143–146]. Of note, ER stress-induced necroptosis is negatively affected by caspase-8 [144]. Additionally, ER stress-triggered necroptosis is mediated by the recruitment of cathepsin B-associated activating protein 1 (AP-1) [146]. ER is also involved in necroptosis feedback loops. ER stress-mediated activation of MLKL reciprocally aggravates ER stress via sensors like PERK (protein kinase R-like ER kinase), IRE1α (inositol-requiring enzyme 1 α), and ATF6 (activating transcription factor 6) to further mediate extracellular signaling between necroptotic cells and the microenvironment through RNA released in extracellular vesicles [147].

Furthermore, there is some evidence that Golgi apparatus participates in intracellular MLKL trafficking in necroptosis [148].

It is important to mention that RIPK3 and MLKL, which are critical for necroptosis, continuously shuttle between the cytosol and nucleus. Therefore, inhibitors of nuclear export have been shown to block cell death [149]. Despite the fact that nuclear translocation of MLKL is not essential for execution of necroptosis, it can still facilitate this RCD [150]. Notably, nuclear translocation of MLKL is reported to stimulate necroptosis in a RIPK1/RIPK3-independent manner [151].

To sum up, multiple subcellular sites are involved in necroptosis either as signaling hubs frequently acting both upstream and downstream of the RIPK1/RIPK3/MLKL axis forming feedback loops. However, organelle-specific mechanisms are highly likely dispensable for necroptosis execution.

## Necroptosis in the erythroid lineage

The number of studies focusing on the contribution of necroptosis or components of its machinery to erythropoiesis is limited (Fig. 2). Initial studies reported a crucial role of RIPK1 for hematopoiesis in general and for erythropoiesis in particular, since its deficiency is associated with anemia [152]. However, it can be assumed that this effect is not mediated by necroptosis, since RIPK3 and MLKL are not essential for normal hematopoiesis [153]. On the other hand, RIPK3 has been shown to regulate hematopoiesis in stress conditions by triggering senescence of HSCs and by promoting MLKL-dependent necroptosis [153, 154].

Importantly, the occurrence of necroptosis has been experimentally demonstrated in murine erythroid precursor cells. Of note, glutathione peroxidase 4 (GPX4)-deficient cells in the erythroid lineage were found to be more prone to RIPK3-mediated necroptosis, which was found to be ROS-dependent. At the same time, necroptosis in anemic GPX4-deficient mice was TNF-α-, CD95-, or PARP (poly (ADP-ribose) polymerase)-independent, suggesting a cell type-specific nature of this necroptosis [155]. The importance of antioxidant GPX4 and ROS signaling in necroptosis of erythrocyte precursors has been attributed to an oxidative environment linked to the accumulation of hemoglobin [156]. The erythroid cells-specific RIPK3-dependent necroptosis complex [155] seems to be different from conventional necroptosis. Erythroid cells contain RIPK1, RIPK3, and caspase-8 but lack FADD [156]. Although GPX4 is primarily protective in ferroptosis, its contribution to regulation of apoptosis, necroptosis, pyroptosis, and parthanatos has been described [157].

It is important to note that during hematopoiesis, necroptosis can occur as a backup to apoptosis. Mice with *Bax*,* Bak*, and *Bid* triple-knockout showed inhibition of apoptosis associated with necroptosis induction. Notably, necroptosis was found to be RIPK1-dependent, incited inflammation in the bone marrow, and promoted ineffective hematopoiesis. As for the erythroid lineage, further RIPK1 inactivation normalized the number of progenitor cells in the bone marrow and peripheral RBC count [158].

Even though mechanisms driving and regulating the necroptotic cell death machinery in the erythroid lineage are not yet fully clarified, it seems clear that there are certain cell type-dependent features. Defining these features may help provide a greater understanding of erythropoiesis and reveal molecular targets to regulate this process pharmacologically.

## The cellular complexity-related features of ferroptosis: the role of organelles

Ferroptosis was first described in 2012. It represents an iron-driven lipid peroxidation-mediated RCD which has recently drawn wide attention in cancer research due to its contribution to tumorigenesis as well as in relation to the opportunity to target it pharmaceutically as a cancer treatment strategy [159, 160]. Ferroptosis requires ROS-mediated peroxidation of specific phospholipids and prevention of their elimination. This is achieved via activation of acylcoenzyme A (CoA) synthetase long-chain family member 4 (ACSL4) and lysophosphatidylcholine acyltransferase 3 (LPCAT3) required to incorporate polyunsaturated fatty acids (PUFAs) into phospholipids. Additionally, ferroptosis is facilitated upon inactivation of the antioxidant enzyme GPX4 [161]. In addition to the reduced glutathione (GSH)/GPX4 axis, ferroptosis is inhibited by the ferroptosis suppressor protein 1 (FSP1)/coenzyme Q10 (CoQ10), GTP cyclohydrolase 1 (GCH1)/tetrahydrobiopterin (BH4), and dihydroorotate dehydrogenase (DHODH)/CoQH2 pathways [162]. Iron triggers the Fenton reaction and associated ROS production, which, in turn, triggers ferroptosis-promoting lipid peroxidation [163].

Importantly, it has been demonstrated that lipid peroxidation in ER is an essential event in ferroptosis. It implies that ER is required for execution of this RCD [164]. Additionally, mitochondria are important regulators of ferroptosis. In ferroptosis, mitochondria undergo characteristic morphological alterations including formation of more dense membranes, volume reduction, and loss of cristae [165]. Mitochondria affect ferroptosis at the level of the cytosolic iron pool regulation by fine-tuning the iron influx from the cytosol into mitochondria, at the level of electron transport chain by adjusting mitROS generation and oxidative phosphorylation via AMPK activation in the energy-deficient state, and at the level of tricarboxylic acid (TCA) cycle affecting the content of NADPH and GSH [162, 166].

The role of other organelles in ferroptosis is much less studied. However, an accumulating body of evidence indicates that other organelles like lysosomes, the Golgi apparatus, and nucleus are involved in ferroptosis execution. For instance, lysosomes can regulate the iron pool and adjust iron levels by ferritin degradation [167]. Furthermore, ferroptosis requires STAT3-mediated expression of lysosomal protein cathepsin B [168] and a recent study demonstrated the role of lysosomal cystine in modulation of sensitivity to ferroptosis [169]. The Golgi apparatus regulates cellular redox homeostasis and consequently prevents ferroptosis. Therefore, Golgi stress induces this iron-dependent RCD presumably via the prooxidant/antioxidant balance impairment [170]. Interestingly, the nucleus affects susceptibility to ferroptosis by engaging transcription factors that might regulate expression of ferroptosis-regulating proteins [171].

To sum up, multiple cellular organelles affect iron accumulation and iron-driven Fenton reaction-mediated ROS production, which are considered crucial hallmarks of ferroptosis. It suggests that there is a wide ferroptosis machinery. Notably, organelles-mediated mechanisms seem to play an important and multifunctional role in ferroptosis execution and mediate the crosstalk between ferroptosis and other RCDs like apoptosis [172].

## Ferroptosis in erythropoiesis

Several studies have investigated the occurrence of ferroptosis in bone marrow precursors of erythrocytes and explored the role of this RCD in hematopoiesis in general and specifically in erythropoiesis (Fig. 2). For instance, experimental evidence suggests that embryonic erythropoiesis requires ferroptosis. Its physiological role in embryonic erythropoiesis is still not fully understood, but there is some evidence that it might participate in differentiation of different hematopoietic lineages. Nevertheless, it is clear that embryonic nucleated erythrocytes can undergo this RCD [173]. Zhao et al. showed that HSCs were prone to ferroptosis due to reduced protein synthesis rates resulting from MYSM1 (Myb-like SWIRM and MPN domains 1) deficiency. MYSM1 is a histone deubiquitination-mediating transcriptional regulator and its deficiency alters gene expression of iron metabolism-regulating proteins, which promotes iron accumulation, and downregulates expression of the protective *GPX4* gene [174]. Similarly to other cell types, ferroptosis of HSCs is lipid peroxidation-dependent and can be attenuated by antioxidant tocopherols and GPX4 [175]. Of note, ferroptosis likely contributes to unbalanced myeloid/lymphoid lineage differentiation of long-term HSCs via cholesterol-mediated promotion of ferroptosis resistance in a SLC38A9–mTOR axis-dependent fashion in a murine model of hypercholesterolemia. This regulatory effect of ferroptosis is achieved by adjusted expression of pro-ferroptotic SLC7A11 and anti-ferroptotic GPX4, as well as ferritinophagy [176]. Moreover, ferroptosis has been shown to inhibit megakaryocytopoiesis in an iron-driven manner [177]. Since MEPs are shared precursors for the megakaryocyte/erythroid lineages, ferroptosis might regulate specification of these cells. In orthochromatic erythroblasts, GPX4 (a critical anti-ferroptotic enzyme) is required for enucleation [178]. However, this effect seems to be ferroptosis-independent. Notably, high expression of GPX4 in erythroblasts likely confers ferroptosis resistance in these cells [178]. Furthermore, GPX4 contributes to mitochondrial clearance in precursors of erythrocytes and GPX4-deficient erythropoiesis is inefficient and associated with abnormal reticulocyte maturation [179].

Notably, iron overload, ROS accumulation, abnormal ferroptosis, and inefficient erythropoiesis are all hallmarks of β-thalassemia [180].

Taken together, ferroptosis and individual components of its machinery are implicated in erythropoiesis at its various stages and could contribute towards the necessary balance between differentiation of different hematopoietic lineages. However, it is important to note that although it is widely accepted that ferroptosis critically contributes to erythropoiesis, more studies are necessary to unveil the distinct role of ferroptosis in normal and altered erythropoiesis.

## Cell death in mature erythrocytes

Cell death of erythrocytes has long been considered a semantic issue due to the undefined status of RBCs, which are not actual organelles-containing cells [118]. Moreover, in the recent NCCD guidelines, the term *eryptosis* is suggested to be avoided, which is explained by the questionable status of erythrocytes as entities between life and death [6]. Early studies have demonstrated that staurosporine, a non-selective inhibitor of kinases and well-known inducer of apoptosis, as well as serum deprivation don’t trigger cell death in mature erythrocytes [181]. However, at the same time, RBCs have been shown to possess a functional caspase network, including the major initiator caspase-8 and executioner caspase-3 [181–183]. However, common pro-apoptotic stimuli do not activate these caspases [182]. Indeed, a mounting number of studies have demonstrated that RCD of erythrocytes can occur and that it is most frequently executed without recruitment of caspase-3 [19]. This difference in comparison to apoptosis might be related to the fact that RBCs don’t possess apoptotic proteins upstream of caspase-3 like cytochrome C, Apaf-1, caspase-2, caspase-6, caspase-7, or caspase-9 [181–183]. On the other hand, ionomycin, a Ca^2+^ ionophore, triggers RBC shrinkage, phosphatidylserine externalization, and cell membrane blebbing, i.e. alterations typical for apoptotic cells [184]. This cell death mode of erythrocytes was suggested to be termed eryptosis in 2005 [185]. Since then, it has been revealed how Ca^2+^ signaling orchestrates cell death of erythrocytes and mediates the above-outlined morphological changes. Ca^2+^ signaling-associated phospholipid membrane scrambling is mediated by upregulation of scramblase and downregulation of flippase, which consequently leads towards the observed asymmetry with phosphatidylserine externalization [186]. Additionally, Ca^2+^ overload induces K^+^ efflux through Gardos channels [187] and promotes activation of cytoskeleton-degrading calpain [188], which consequently leads to the cell shrinkage and membrane blebbing, respectively. In its turn, Ca^2+^ accumulation in erythrocytes is promoted by shear stress, oxidants, ceramide, prostaglandin E2, PKC, CK1α, or p38 MAPK [16, 19, 187].

Importantly, Berg et al. reported that mature erythrocytes lacked Bcl-2, as well as other anti-apoptotic proteins such as XIAP, c-IAP1, and c-IAP2 [182]. Conversely, it has been shown that mature erythrocytes contain Bcl-XL and Bak, but don’t express cytochrome c, Bax, or Bad [189]. Despite the described role of pro-apoptotic BH3-only proteins in reticulocytes, there is no evidence that mature RBCs contain them. At the same time, Bak-derived BH3 peptide can trigger cell death in erythrocytes [189]. In-depth understanding of the content and role of the Bcl-2 protein family members, which are major orchestrators of apoptosis, is crucial for uncovering cell death mechanisms in RBCs [190].

**Fig. 3:**
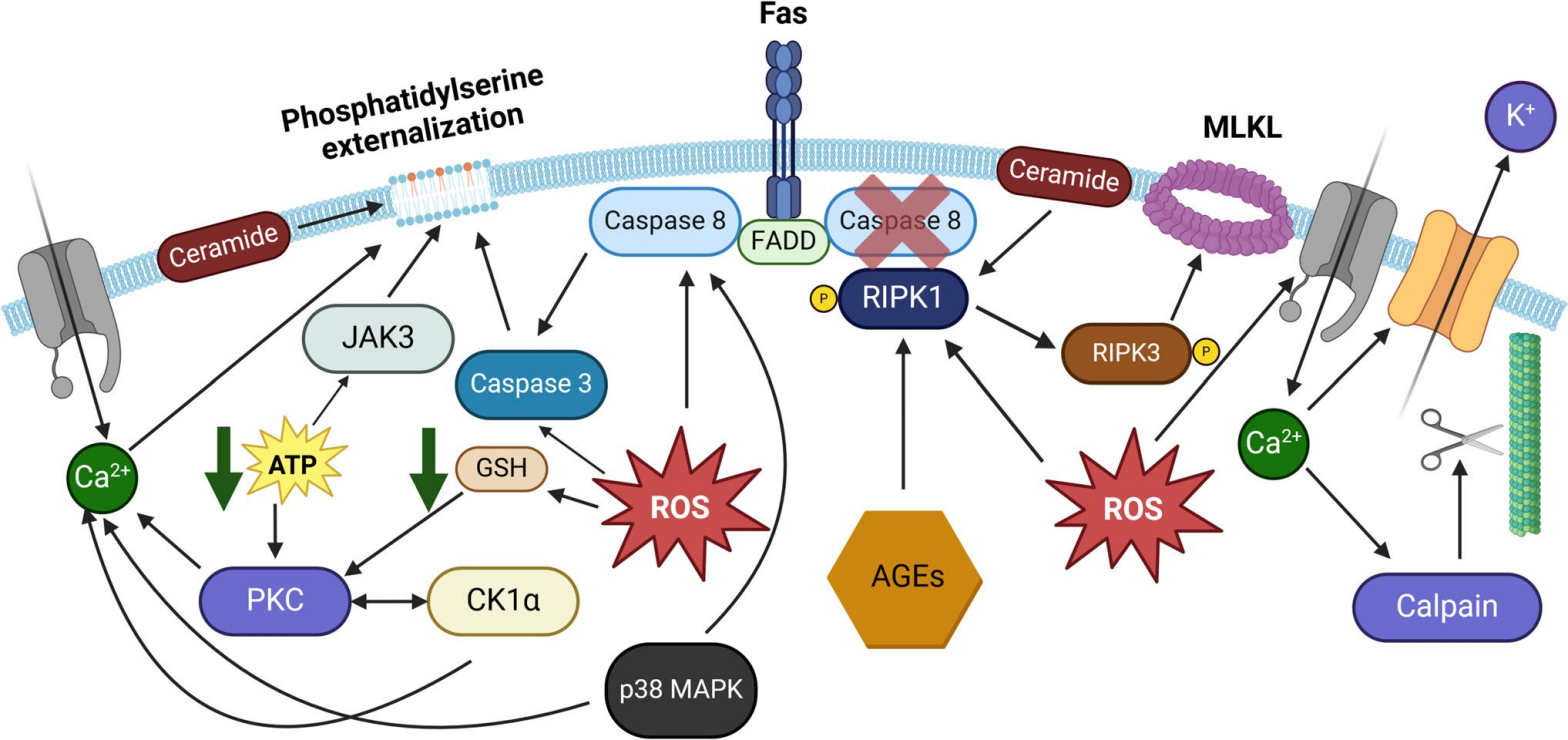
Cell death signaling in mature erythrocytes. Mature erythrocytes can undergo eryptosis and necroptosis. Eryptosis relies on Ca^2+^ signaling, which mediates phosphatidylserine activation, activation of cytoskeleton-degrading calpain, and K^+^ efflux through Gardos channels to promote cell shrinkage, whereas necroptosis is mediated by the RIPK1/RIPK3/MLKL signaling. Ceramide and ROS are signaling mediators of both pathways. Notably, the caspase-8 status and factors that regulate its activation (like ROS) might determine a cell death mode preference, switching from necroptosis to eryptosis (if active). *AGEs* – advanced glycation end products; *CK1α* – casein kinase 1 α; *FADD* – Fas-associated protein with death domain; *GSH* – reduced glutathione; *JAK3* – Janus kinase 3; *MLKL* – mixed lineage kinase domain like pseudokinase; *P* – phosphate group; *p38 MAPK* – p38 mitogen-activated protein kinase; *PKC* – protein kinase C; *RIPK1* – receptor-interacting serine/threonine-protein kinase 1; *RIPK3* – receptor-interacting serine/threonine-protein kinase 3; *ROS* – reactive oxygen species

Mature erythrocytes have been shown to retain components of the extrinsic apoptosis machinery, including Fas, FasL, and FADD [191]. Although Berg et al. reported in an early study that Fas signaling stimulation did not lead to caspases recruitment in erythrocytes [182], it was later demonstrated that phosphatidylserine translocation to the outer membrane in RBCs was regulated by the assembly of the Fas/FADD/caspase-8 complex and downstream activation of caspase-3 [191]. On the other hand, it is not clear whether FasL induces the formation of this complex, since it was reported that its activation was ROS-dependent and ligand-independent due to the inability of Fas agonists to activate caspase-8 and trigger cell membrane scrambling in mature RBCs [192]. Contribution of ROS-mediated Fas signaling to the cell death of mature erythrocytes has been confirmed in additional studies [193]. However, a recent study by Restivo et al. has clearly demonstrated that Fas-associated death-inducing signaling complex (DISC) comprising FADD and caspase-8 can be ROS-independent and p38 MAPK-dependent [194–196]. Thus, Fas-mediated death of erythrocytes was clearly demonstrated and confirmed. On the other hand, little is known about other death receptors, TNF-R1, TRAIL-R1, and TRAIL-R2. Notably, the signaling events downstream of these death receptors as well as Fas are known to trigger a distinct cell death called necroptosis or programmed necrosis [197]. Necroptosis has been demonstrated in mature erythrocytes with an emphasis on the key contribution of the RIPK1/RIPK3/MLKL axis to its regulation [18, 198, 199]. It should be emphasized that necroptosis of mature erythrocytes relies on Fas/FasL signaling but, unexpectedly, it is not induced by TNF-α or TRAIL [18, 199]. There is still only a limited number of studies that have attempted to uncover the mechanisms governing necroptosis in erythrocytes, but there is evidence that necroptotic signaling in erythrocytes requires pore formation, necrosome assembly, NADPH oxidase- and Fenton chemistry-derived ROS, glucose-derived advanced glycation end products, ceramide, and Syk-mediated Band 3 protein phosphorylation [17, 198–201]. Thus, it turned out that erythrocytes have a more complex functional cell death system than we used to think (summarized in Fig. 3), albeit less diverse than in its precursors.

Is the interplay between eryptosis and necroptosis of mature erythrocytes possible? If so, erythrocytes are capable of cell fate decision making, which challenges the view that they are post-apoptotic preserved organelle-free entities [118]. Of note, eryptosis and necroptosis in erythrocytes are frequently mutually exclusive [18], which might indicate the presence of specific cell fate decision points. In particular, vaginolysin [198], intermedilysin [198], and high glucose concentrations combined with pore-forming toxins [200] induce necroptosis of erythrocytes at concentrations that don’t induce eryptosis. At the same time, eryptosis is induced by efavirenz [202], NSC-95397 [203], N, N-diethyl-3-methylbenzamide (DEET) [204], gingerol [205], bioymifi [206], and nickel chloride [207] at concentrations that don’t trigger erythronecroptosis. To our knowledge, simultaneous induction of both lethal subroutines of erythrocytes by the same agent has not been reported. Notably, triclosan induced eryptosis, which could be ameliorated by necrostatin-1, a RIPK1 inhibitor [208]. However, MLKL was not involved in triclosan-induced cell death of erythrocytes, probably excluding necroptosis as a cause of death [208], since the NCCD defines necroptosis as an MLKL-dependent RCD [6]. Valuable insights into the molecular mechanisms of these decision points and the associated crosstalk could be provided by the analysis of links shared by both pathways. Primarily, it is logical to consider caspase-8 as a possible molecular switch that determines the plasticity of cell death pathways in mature erythrocytes, since caspase-8 is a well-known cell fate-determining enzyme, regulating apoptosis, necroptosis, or pyroptosis depending on its own activation status and interactions with other components of the cell death machinery [129, 209]. Notably, caspase-8 has been shown to be a component of the eryptosis-regulating FasL/FADD/caspase-8 complex [196] and the RIPK1/FADD/caspase-8 necroptosis-regulating necrosome in erythrocytes [198]. Moreover, caspase-8 was found to antagonize necroptosis, as evidenced by accelerated necroptosis of erythrocytes in response to caspase-8 inhibition [198]. Thus, caspase-8 might be at the crossroads of cell death pathways in erythrocytes like in nucleated cells. At the moment, experimental evidence of this hypothesis is rather limited and it is supported only by the pioneering study of LaRocca et al. [198]. Given the minor contribution of the caspase-8/caspase-3 axis to eryptosis, it can be speculated that these components of cell death machinery are retained in mature erythrocytes to ensure execution of immunologically silent eryptosis, avoiding lytic and potentially highly immunogenic necroptosis. The functional caspase network in mature erythrocytes cannot be considered just a heritage of the erythroid maturation process but a component of the cell fate decision system, which can be involved in the regulation of the immune response by determining a cell death mode (lytic pro-inflammatory necroptosis vs. non-lytic anti-inflammatory eryptosis). However, more studies are necessary to provide experimental evidence for this hypothesis. Moreover, ROS are generally accepted as significant players in the crosstalk between cell death pathways [210, 211]. Contribution of ROS to both eryptosis and necroptosis of erythrocytes has been recently summarized [17]. Of note, ROS may inactivate caspase-8 to regulate apoptosis/necroptosis [201]. On the other hand, ROS were reported to be essential for the activation of the FasL/FADD/caspase-8/caspase-3 axis and for the phosphatidylserine externalization [191]. In general, ROS have been shown to mediate the shift from apoptosis to necroptosis in nucleated cells by inhibition of caspases and activation of necroptosis-associated kinases [212]. Thus, the redox status of erythrocytes might be an important factor in determining whether a red blood cell dies via eryptosis or necroptosis.

Dreischer et al. have provided a link between eryptosis and ferroptosis in mature erythrocytes, suggesting the presence of certain overlaps [213]. Indeed, hemoglobin of erythrocytes contains the bulk of intraorganismal iron [214]. Additionally, erythrocytes generate ROS due to hemoglobin autooxidation, Fenton reaction, NADPH oxidase- and xanthine oxidoreductase-mediated reactions, and scavenge exogenous immune cells-derived ROS [17]. Moreover, the redox-sensitive Keap1-Nrf2 pathway is activated in nucleated cells following oxidative stress to provide an adaptive and protective response by antioxidant enzymes upregulation [215], which is not possible in mature erythrocytes.

Calcium signaling has been found to be a hallmark of ferroptosis, suggesting that Ca^2+^ is a master regulator of this RCD [216]. Given the role of calcium signaling in eryptosis, it could represent another important link between both RCDs in erythrocytes. Iron abundance, proneness to oxidative stress, the role of Ca^2+^ signaling in cell death, and the capability of other erythroid lineage cells to undergo ferroptosis all together indicate that this iron-driven cell death pathway might exist in RBCs. However, no studies have confirmed that mature erythrocytes can undergo ferroptosis so far. It is important to note that confirmation of ferroptosis in erythrocytes can be challenging due to the impossibility to assess characteristic alterations of organelles (especially mitochondria, absent in mature RBCs) and the complex ferroptosis signaling requiring epigenetic, transcriptional, and post-translational regulation. The lack of specific ferroptosis markers drives the necessity to determine ferroptosis in a complex way, which includes morphological, biochemical, and genetic hallmarks as well as evaluation of degradation and upregulation of certain proteins [217]. This could be very difficult, if possible at all, in the case of erythrocytes. It suggests that it is impossible to fully detect and recognize the occurrence of ferroptosis in RBCs at the current level of our erythrocyte-related knowledge.

On the other hand, studies of the ferroptosis machinery in mature erythrocytes can provide valuable insights. Notably, circulating RBCs contain GPX4, which is functionally active and was reported to prevent hemolysis upon RBC storage [218]. Iron accumulation in RBCs causes ROS production and damage to macromolecules [219]. Like in ferroptosis, erythrocytes frequently experience ROS overgeneration and depletion of GSH and antioxidant enzymes in response to xenobiotics [220]. In addition, metabolomic and genomic studies have revealed that polymorphisms of ferroptosis-associated *LPCAT3* are connected with different levels of lysophosphatidylserines in erythrocytes [221]. Another marker upregulated and accumulated in ferroptosis is transferrin receptor (TfR1) [222]. It is detected in reticulocytes but upon maturation its content decreases and it is absent in mature erythrocytes [223]. These studies suggest that erythrocytes have retained some ferroptosis-associated factors. Unfortunately, multiple components of the ferroptosis machinery are not investigated and characterized in mature erythrocytes. It is still not clear whether erythrocytes can undergo a distinct iron-driven cell death or whether iron-mediated mechanisms contribute to execution of eryptosis. We believe that high-quality proteomic studies might shed light on the availability of cell death machinery-related proteins in mature RBCs, which will help unravel the involved molecular mechanisms.

## Clinical applications of erythrocyte cell death

Optimization of RBC preservation efficacy remains one of the urgent challenges in blood transfusion [224]. Hemolysis of erythrocytes has long been recognized as a key storage-induced lesion [225]. However, Lang et al. demonstrated that RBC storage enhanced susceptibility to eryptosis as well, showing that the lifespan of transfused stored erythrocytes was shorter in comparison to intact cells in a murine model [226]. Moreover, RBCs with exposed phosphatidylserine were shown to negatively affect endothelial cells, triggering microcirculation disturbances [226]. Since eryptosis of stored RBCs is mitigated by EPO, this can provide novel insight into enhancing the erythrocyte preservation efficacy. Likewise, storage increases the susceptibility of RBCs to necroptosis via NADPH oxidase-derived ROS-dependent RIPK1 activation [201]. Thus, eryptosis and erythronecroptosis cannot be neglected within quality assessment of stored RBCs intended for transfusion.

Notably, accumulating evidence suggests that both eryptosis and erythronecroptosis might be involved in the host defense against pathogens. In particular, *P. falciparum* is known to induce eryptosis, which is believed to be a double-edged sword in malaria. On one hand, it eliminates infected cells. *Plasmodium* parasites have evolved the mechanisms to sequester Ca^2+^ to prevent eryptosis [227]. Furthermore, protective effect of eryptosis in malaria (which is enhanced in non-infected cells as well) has been linked to the control of parasitemia due to the insensitivity of eryptotic cells to the pathogen [228]. On the other hand, phosphatidylserine exposure in malaria-infected eryptotic cells promotes interactions with endothelial cells with the negative impact on their function [229]. Moreover, it is suggested that the deficient immune response in malaria might be at least partly attributed to enhanced efferocytosis of eryptotic cells by macrophages [227]. Additionally, eryptosis has been linked with the response to *S. pneumoniae*, a common causative agent of lower respiratory infections. In particular, eryptosis of erythrocytes under the influence of *S. pneumoniae-*derived pneumolysin prevents hemolysis [230]. At the same time, *G. vaginalis*- and *S. intermedius*-derived bacterial pore-forming toxins can trigger necroptosis of erythrocytes, which is suggested as a bacterial strategy to promote their growth [198]. Accelerated eryptosis has also been shown in COVID-19. Its enhancement has been attributed to elevation of pro-inflammatory cytokines and linked to a high thrombotic burden in COVID-19 patients [231]. Thus, converging lines of evidence show that RCDs of erythrocytes are important players in protozoan, bacterial and viral infections, suggesting a possible benefit of RCDs-directed pharmacological interventions.

Abnormalities of RCD pathways in mature erythrocytes are well-documented for inherited blood disorders. Increased erythrocyte clearance has been associated with eryptosis in thalassemia [232], hereditary spherocytosis [232], sickle cell disease [233], and glucose-6-phosphate dehydrogenase deficiency [234]. The susceptibility of erythrocytes to eryptosis in glucose-6-phosphate dehydrogenase deficiency was attributed to oxidative stress [234], while in sickle cell disease Ca^2+^ entry was reported to be facilitated by ceramide [233]. Studies focusing on lethal subroutines in genetic blood disorders expand our knowledge of their pathogenesis and open novel therapeutic avenues for reducing anemia in these conditions.

Hemolysis tests are widely used to assess hemocompatibility during drug development [235]. In addition, other RBC mechanical stability tests, like the Couette type ektacytometer, have been suggested to detect sub-hemolytic damage [236]. Evaluation of eryptosis has also been shown as a strategy to reveal sub-hemolytic cytotoxic effects against erythrocytes during development of nanomaterials-based drugs [220]. However, this approach seems to be valid beyond nanotoxicology. In particular, anti-cancer chemotherapy is frequently complicated by anemia [237]. Accumulating evidence indicates that chemotherapy-induced anemia can be linked with enhanced eryptosis [238]. Assessment of eryptosis thus might improve the overall evaluation of cytotoxicity of potential chemotherapeutic agents to predict their ability to promote anemia as a side effect.

**Fig. 4:**
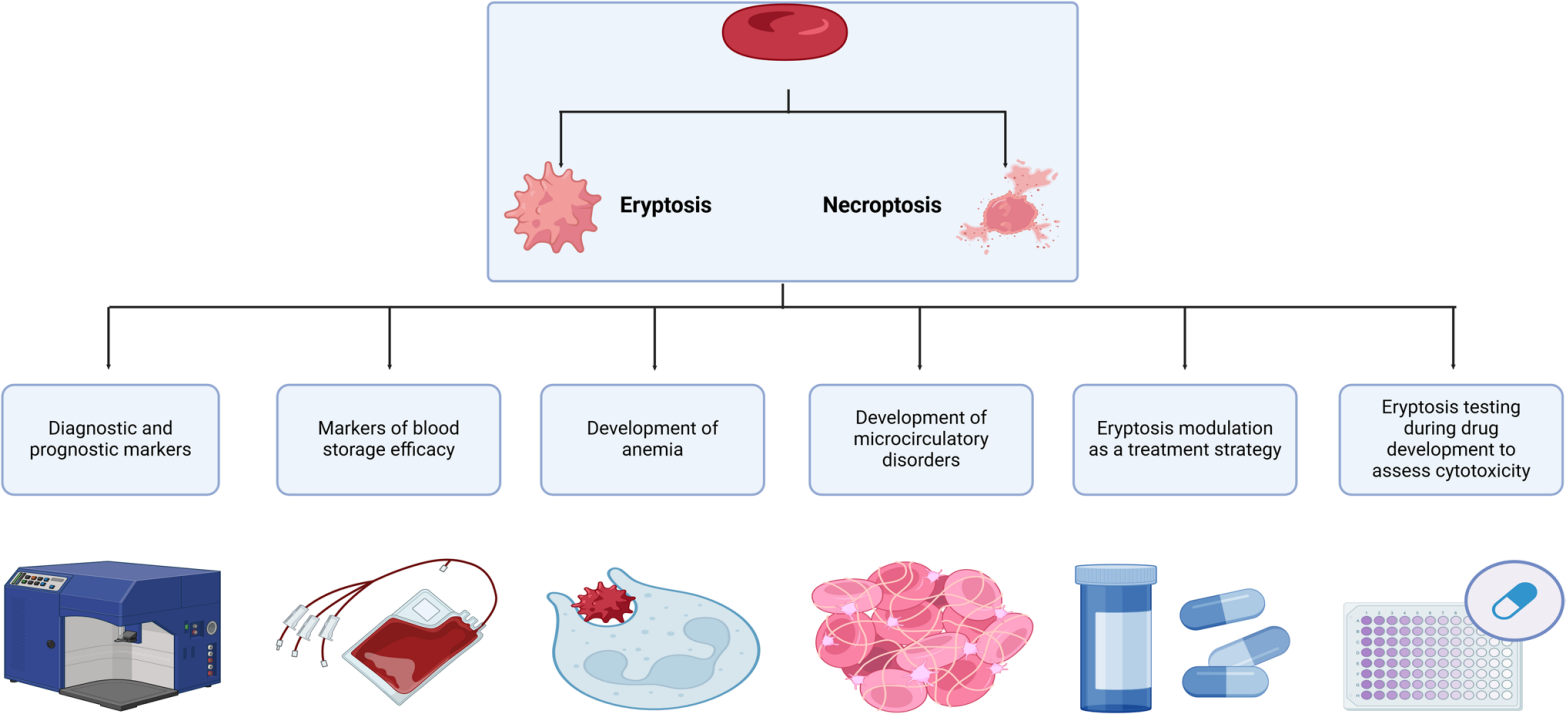
Clinical applications of erythrocyte regulated cell death pathways. Eryptosis and erythronecroptosis, as two forms of erythrocyte regulated cell death, can be applied as markers of red blood cell storage efficacy. Clinical applications of eryptosis are better investigated than those of erythrocyte necroptosis. Eryptosis is of diagnostic and prognostic significance in multiple diseases. Enhanced eryptosis contributes to anemia, endothelial dysfunction, and formation of microclogs. Eryptosis modulation is a promising therapeutic strategy to prevent the above-mentioned eryptosis-associated complications. Eryptosis is a promising approach to test the cytotoxicity of potential drugs

The rising awareness of the role of accelerated eryptosis in various diseases is well summarized in multiple recent reviews [239–241] and has attracted interest towards diagnostic potential of eryptosis parameters. For instance, eryptosis is suggested as an early biomarker of potentially upcoming acute complications in sickle cell disease [233]. Diagnostic potential of eryptosis has been widely studied in diabetes mellitus (DM). For instance, determination of caspase-3-positive erythrocyte count was suggested as a tool to diagnose functional anemia in DM [242]. Moreover, it is known that glycated hemoglobin triggers eryptosis in DM [243]. However, diagnostic links between eryptosis parameters and glycated hemoglobin are not fully clear. Contribution of eryptosis to microthrombosis is well reported and it is also reflected in the study by Katahira et al., which suggests to use eryptosis parameters as markers for disseminated intravascular coagulation [244]. Diagnostic prospective of eryptosis has been appreciated in neurological diseases as well. Eryptosis was tested as a biomarker of the severity of Parkinson’s disease [245]. Furthermore, in aneurysmal subarachnoid hemorrhage, enhanced eryptosis in the cerebrospinal fluid is a prognostic marker for developing complications such as delayed cerebral ischemia [246]. Thus, eryptosis parameters seem to be of potentially important diagnostic and prognostic significance and further research in this field should be encouraged (Fig. 4).

## Organelle clearance during maturation of erythrocytes is associated with the loss of components of the cell death machinery

A growing number of studies indicate that mature erythrocytes have a precisely structured RCD machinery comprising of several lethal subroutines with clearly defined molecular mechanisms. Biochemical hallmarks of eryptosis include Ca^2+^ overload, phosphatidylserine externalization, ceramide accumulation, and ROS overproduction, while necroptosis of erythrocytes requires formation of the necrosome complex (RIPK1/RIPK3/MLKL). Several recent discoveries have shown that there are certain overlaps between eryptosis and erythronecroptosis, which occur in an orchestrated manner and are often repugnant. Therefore, erythrocytes might be capable of determining the way they die depending on the circumstances. Our analysis supports the notion that caspase-8 and ROS may act as the platforms regulating the interplay between eryptosis and erythronecroptosis. Multifaceted effects of ROS and multiple downstream targets of ROS indicate that resulting consequences might be stimulus-dependent. ROS could determine a cell death mode in a concentration-dependent way. The existence of overlapping signaling pathways suggests that possible therapeutic interventions might be explored with the aim to favor one RCD over the other, since inhibition of one RCD may lead to activation of the other one. Importantly, apoptosis and necroptosis of nucleated cells have opposite immunogenic effects (anti-inflammatory and pro-inflammatory, respectively) [247]. The discovery of erythronecroptosis should, therefore, drive research on immunogenicity of erythrocyte cell death. RBC death might play an important role in regulation of immune response and immune surveillance, since it has become clear that necroptosis of nucleated cells promotes inflammation by necroptotic cells-derived damage-associated molecular patterns (DAMPs) [248]. Moreover, the abundance of erythrocytes in circulation [23] raises an additional question concerning the possible immunogenic consequences of erythrocyte RCDs. Even though more studies are required to provide in-depth insight into immunogenicity of erythrocyte cell death modalities, it can be assumed even now that their targeting can be beneficial. Moreover, we believe that the caspase-8-mediated crosstalk between eryptosis and erythronecroptosis is one of the reasons why the caspase machinery is retained in mature erythrocytes. As outlined above, the apoptotic machinery is of paramount importance in the erythroid lineage to ensure balanced erythropoiesis. Furthermore, caspases not only mediate apoptosis but are also required for survival and differentiation of erythroid cells. Mitochondrial clearance results in the loss of multiple regulators and effectors of the intrinsic apoptotic pathway. On the other hand, the reason why Fas/FasL signaling, caspase-8, and caspase-3 are retained in mature erythrocytes could be to provide the interplay between eryptosis and necroptosis of erythrocytes. This hypothesis is supported by the data that Fas-mediated cell death in mature erythrocytes can be triggered by internal stimuli like ROS and p38 MAPK signaling [194–196]. Moreover, caspase-3 and caspase-8 are rarely recruited for execution of eryptosis [19], which suggests that they might have another function in mature erythrocytes.

It is important to note that CK1α triggers eryptosis in mature erythrocytes [16, 249]. At the same time, CK1α is protective against apoptosis and mediates anti-apoptotic effects of EPO during erythropoiesis [107]. EPO has been shown to exert the anti-eryptotic effect in mature erythrocytes via ROS reduction [250]. The opposite effect of CK1α in erythroid precursors and mature RBCs might be explained by the lack of the caspase-10-Bid axis in erythrocytes, which is at least partly responsible for anti-apoptotic action of CK1α in the erythroid precursors. At the same time, CK1α mediates Ca^2+^ influx in mature erythrocytes, an effect that is no more counterbalanced by an anti-apoptotic influence of CK1α [251].

It should be also noted that EPO- and SCF-induced erythroid differentiation is associated with upregulation of PKC-α [252]. Moreover, PKC-ε protects erythroid cells from TRAIL-induced apoptosis via Bcl-2 levels modulation [253]. In non-erythroid cells, PKC-ε has also been reported to downregulate pro-apoptotic Bid [254]. Taken together, anti-apoptotic effects of PKC are associated with its ability to regulate the expression of anti-apoptotic and pro-apoptotic factors, a mechanism that cannot be achieved in mature erythrocytes. In mature erythrocytes, PKC induces eryptosis by stimulating Ca^2+^ signaling [255, 256]. Thus, like in the case of CK1α, the opposite effect of PKC in erythroid precursors and mature erythrocytes can be explained by the lack of the anti-apoptotic machinery through which PKC can exert its anti-apoptotic action. Instead, PKC promotes Ca^2+^ influx, which mediates PKC-induced eryptosis.

In the erythroid lineage, p38 MAPK is upregulated in response to EPO signaling and is necessary for further EPO-triggered differentiation [257]. Intriguingly, p38 MAPK is known to induce eryptosis [258]. Thus, the effect of CK1α, PKC, and p38 MAPK varies from pro-survival in some erythrocyte precursors to pro-eryptotic in mature cells, which might be a consequence of missing downstream effectors in circulating erythrocytes.

The intrinsic MOMP-associated apoptotic pathway is tightly regulated by the Bcl-2 family of proteins comprising anti-apoptotic Bcl-2, Bcl-XL, Bcl-W, pro-apoptotic effector Bax and Bak proteins, and BH3-only proteins, including Bad, Bim, PUMA, and Bid (modulating apoptosis through antagonizing anti-apoptotic Bcl-2 or inducing the activity of Bax or Bak) [80, 259, 260]. There is scarce evidence that mature erythrocytes could contain some members of the Bcl-2 family of proteins. However, in erythrocytes, Bcl-XL and Bak are membrane-associated, the total intracellular amount is lower than in nucleated cells, and they are involved in caspase-independent cell death regulation by promoting Ca^2+^ overload. Moreover, Bcl-XL was shown to be protective against Bak-derived BH3 peptide-induced cell death in mature erythrocytes [189]. The functional Bcl-XL/Bak system (sensitive to regulation via BH3 proteins) is still retained in erythrocytes and instead of triggering MOMP, it affects Ca^2+^ signaling crucial for erythrocyte cell death. It is important to note that in nucleated cells, Bcl-XL inhibits caspase-8 with consequent switch of the cell death pathway towards necroptosis [261]. Thus, it is tempting to speculate that its expression in mature erythrocytes might be also associated with selection of a cell death pathway.

Since there are no lysosomes in erythrocytes, lysosome-associated cell death machinery in these cells is poorly studied. Of note, erythrocytes were found to contain cathepsin E, a mediator of peroxynitrite-induced cell death [262]. Additionally, several early studies demonstrated the presence of cathepsin D in human RBCs [263, 264]. There is no evidence that other cathepsins are found in RBCs. As discussed above, erythrocytes don’t express Bid, Bcl-2, XIAP, which are substrates for cathepsins during modulation of apoptosis. Like in the case of lysosomes, erythrocytes have no ER, Golgi apparatus, and nucleus. Therefore, they lack specific organelle-associated proteins involved in regulation of cell death. Moreover, nucleus and mitochondria contain multiple substrates for caspase-3 [265], which can also provide insight into the non-essential contribution of caspase-3 to eryptosis, since there are no downstream targets of caspase-3 in erythrocytes due to organelle clearance.

Thus, it could be assumed that maturation of erythrocytes is associated with the loss of cell death machinery components and that the retained cell death pathways could be considered a heritage of the erythroid maturation. However, the simplified cell death pathways still contain necessary components to ensure execution and interplay between distinct RCDs, like eryptosis and erythronecroptosis, with potentially opposite immunogenic consequences.

## Conclusions

Emerging evidence suggests that damage to mature erythrocytes might prompt eryptosis or erythronecroptosis execution. Moreover, several points of interconnection can determine the cell fate of erythrocytes. These pathways share some features with their counterparts in nucleated cells but still are characterized by a certain level of uniqueness. The crosstalk between cell death pathways in erythrocytes is still poorly understood. However, the presence of RCD machinery and its cross-regulation indicates that cell death of erythrocytes is not a pure mechanistic disintegration, but rather a regulated process. Upon maturation, erythrocytes lose most organelle-specific cell death responses, which limits the set of available RCD modalities and the regulatory capabilities of cell death machinery. The presence of the functional caspase network (caspase-8 and caspase-3, non-essential for execution of eryptosis) and the Fas cell death signaling pathway could be probably attributed to their ability to mediate the interplay between eryptosis and necroptosis of erythrocytes. Lack of pro-survival downstream effectors for pleiotropic CK1α and PKC might determine their pro-eryptotic role in mammalian red blood cells. Cell death machinery of erythrocytes should be further investigated to explore the possibility of its pharmacological targeting.

## Data Availability

No datasets were generated or analysed during the current study.
